# CLEC5A Promotes Neuronal Pyroptosis in Rat Spinal Cord Injury Models by Interacting with TREM1 and Elevating NLRC4 Expression

**DOI:** 10.1523/ENEURO.0111-24.2024

**Published:** 2024-10-24

**Authors:** Yonghong Tan, Qiong Wang, Yubing Guo, Na Zhang, Yingyi Xu, Xue Bai, Jianhua Liu, Xiaobao Bi

**Affiliations:** Department of Anesthesiology, Guangzhou Women and Children’s Medical Center, Guangdong Provincial Clinical Research Center for Child Health, Guangzhou 510623, China

**Keywords:** CLEC5A, NLRC4, neuronal pyroptosis, spinal cord injury, TREM1

## Abstract

Pyroptosis, an inflammatory programmed cell death, has recently been found to play an important role in spinal cord injury (SCI). C-type lectin domain family 5 member A (CLEC5A), triggering receptor expressed on myeloid cells 1 (TREM1), and NLR-family CARD-containing protein 4 (NLRC4) have been reported to be associated with neuronal pyroptosis, but few studies have clarified their functions and regulatory mechanisms in SCI. In this study, CLEC5A, TREM1, and NLRC4 were highly expressed in lidocaine-induced SCI rat models, and their knockdown alleviated lidocaine-induced SCI. The elevation of pyroptosis-related indicators LDH, ASC, GSDMD-N, IL-18, caspase-1, and IL-1β levels in SCI rats was attenuated after silencing of CLEC5A, TREM1, or NLRC4. Lidocaine-induced decrease in cell viability and the elevation in cell death were partly reversed after CLEC5A, TREM1, or NLRC4 silencing. Lidocaine-mediated effects on the levels of LDH, ASC, GSDMD-N, IL-18, caspase-1, and IL-1β in lidocaine-induced PC12 cells were weakened by downregulating CLEC5A, TREM1, or NLRC4. CLEC5A could interact with TREM1 to mediate NLRC4 expression, thus accelerating neuronal pyroptosis, ultimately leading to SCI exacerbation. In conclusions, CLEC5A interacted with TREM1 to increase NLRC4 expression, thus promoting neuronal pyroptosis in rat SCI models, providing new insights into the role of neuronal pyroptosis in SCI.

## Significance Statement

Pyroptosis has been reported to be involved in SCI. Higher levels of CLEC5A, TREM1, and NLRC4 were associated with neuronal pyroptosis. However, the role and regulatory mechanism of CLEC5A, TREM1, and NLRC4 in SCI were not clear. Here, high expression of CLEC5A, TREM1, and NLRC4 was observed in lidocaine-induced SCI rat models. CLEC5A could interact with TREM1 to enhance the expression of NLRC4, thus accelerating neuronal pyroptosis in rat SCI models. These findings identify CLEC5A, TREM1, and NLRC4 as potential therapeutic targets for SCI.

## Introduction

Spinal cord injury (SCI) is a common global clinical disease that seriously affects the quality of life of patients (Percy and Sabe, 2022). According to reports, ∼10.4–83 people suffer from SCI per 1 million people per year (Cheng et al., 2023). The pathological process of SCI can usually be divided into two major stages: primary injury and secondary injury. Primary injury is an irreversible process that refers to the direct destruction of tissues at the moment of violent trauma, leading to vascular rupture, axonal rupture, and nerve cell death (Klockner et al., 2023). Secondary injury is based on primary injury, causing infiltration of inflammatory cells, ischemia, free radical generation, and release of toxic compounds, triggering a cascade reaction of secondary injury, leading to further permanent injury and neurological dysfunction (Zhu et al., 2023). At present, the commonly used clinical treatment methods for SCI include surgery, glucocorticoid, various neurotrophin and physical rehabilitation, but none of these methods can effectively improve the prognosis (Shen et al., 2023). The neuronal death of SCI is an important link, which is not only the ultimate outcome of various secondary reactions but also the core of inducing the cascade expansion of pathological processes (Lukacova et al., 2021). Therefore, exploring the mechanism of neuronal death after SCI is of great significance.

There are various ways and types of cell death. In SCI, common forms of cell death have been found, such as apoptosis, necrosis, pyroptosis, and autophagy (Z. Shi et al., 2021). Among them, pyroptosis is an inflammation-related programmed cell death, characterized by a strong inflammatory cascade reaction accompanied by the release of a large number of inflammatory factors and cell contents (Yue et al., 2023). In the classical pathway of pyroptosis, the activation of inflammasomes is a critical step. Proteins that can form inflammasomes mainly include typical NLR family proteins, such as NLRP1, NLR-family CARD-containing protein 4 (NLRC4), NLRP3, and so on (Morimoto et al., 2021). A previous reported study revealed that NLRC4/NLRP3 inflammasome-related genes GSDMD, IL-18, and IL-1β were highly expressed in SCI (Shan et al., 2023). The promotion of pyroptosis after SCI by NLRP3 inflammasome has been widely reported (Xiao et al., 2023). Activation of classical NLRC4 inflammasome signaling is related to the binding of NLRC4 to its ligand to form a polyprotein inflammasome complex, which can recruit, cut, and activate caspase-1. Activated caspase-1 is capable of cleaving IL-1β and IL-18 precursors, promoting the release of mature IL-1β and IL-18 (Chebly et al., 2022; Davari et al., 2023). At present, there are few reports on the promotion of neuronal pyroptosis by NLRC4 inflammasomes.

Through biological information analysis (GSE136833 database), we found that C-type lectin domain family 5 member A (CLEC5A), triggering receptor expressed on myeloid cells 1 (TREM1), and were highly expressed in lidocaine-induced SCI models. CLEC5A, a member of the pattern recognition receptor, activates spleen tyrosine kinase (SYK) through the adaptor protein DNAX activating protein 12 (DAP12), thereby activating downstream related signaling pathways and inducing the host to release a large number of cytokines and inflammatory factors such as interleukin-6 (IL-6), tumor necrosis factor (TNF), and interleukin-8 (IL-8; P. K. Chen et al., 2020; Sung et al., 2020). Therefore, CLEC5A is an important participant in human immune response and widely participates in regulating various inflammatory and infectious diseases. Wang et al. reported that CLEC5A knockdown could prevent cardiac dysfunction after myocardial infarction by inhibiting macrophage polarization, NLRP3 inflammasome activation and pyroptosis (de Vasconcelos et al., 2016). Also, TREM1 aggravated chronic obstructive pulmonary disease progression by activating NLRP3 inflammasome and mediating pyroptosis (L. Wang et al., 2021). Furthermore, NLRP12 cooperated with NLRP3 and NLRC4 to promote pyroptosis-induced acute glaucoma ganglion cell death (H. Chen et al., 2020). Therefore, we speculated that CLEC5A, TREM1, and NLRC4 could regulate neuronal pyroptosis after SCI.

Therefore, this study evaluated whether CLEC5A, TREM1, and NLRC4 could mediate neuronal pyroptosis after SCI and the potential mechanisms between them.

## Materials and Methods

### Animals and ethics approval

A total of 30 male Sprague Dawley rats (specific pathogen free) were used in this study, with a body weight of 260–300 g and an age of 3–4 months. The rat was purchased from Animal Center of Southern Medical University (Guangzhou, China). Rats were kept in a controlled environment (22 ± 1°C, humidity 40–60%) with a light/dark cycle of 12/12 h. Rats were given standard food and water *ad libitum* and raised for a week before the experiment to adapt to the environment. The research plans for experimental animals have been approved by the Animal Care and Ethical Standards Animal Experiment Committee of the hospital. This study has been approved by the Ethics Review Committee of Guangzhou Women and Children Medical Center (JENNIO-IACUC-2023-A029).

### Establishment of SCI rat models

All experimental operations were conducted following the ethical treatment guidelines for experimental animals. The L5/L6 spinous process space and ligamentum flavum of rats were exposed through surgery after anesthesia with isoflurane. Subsequently, the PE-10 mini spinal catheter was inserted into the subarachnoid space of the L5/L6 intervertebral space and advanced 2.5 cm toward the cephalad direction. The distal end of the catheter is sealed and subcutaneously fixed. One day after catheter insertion, rats with obvious limb paralysis, motor disorders, or unilateral limb paralysis caused by catheter insertion were excluded. One day after the catheter was inserted, lidocaine (2%, 10 µl) was injected through the catheter into the rest of the rats who showed free activity to verify the position of the catheter tip. This study only used rats that were successfully intubated and showed paralysis in both hindlimbs 30 s after injection.

The experimental rats were randomly divided into five groups with a random number tablet (*n* = 6) as follows: (1) sham, (2) lidocaine, (3) lidocaine + shCLEC5A, (4) lidocaine + shTREM1, and (5) lidocaine + shNLRC4. Lidocaine (10%) was prepared. The lidocaine group received an intrathecal injection of 10% lidocaine (0.12 µl/g body weight), with continuous injection for 3 d, while the sham group received PBS. Lentivirus-mediated sh-NC, shCLEC5A, shTREM1, or shNLRC4 were intrathecally injected into SCI rats with an injection volume of 2 × 10^7^ TU. Fourteen days after the injection of lentivirus, rats were killed to extract spinal cord tissues for subsequent analysis.

### Behavior assessment

The BBB score was used to evaluate the neural function of rats, ranging from 0 to 21 points [0: no significant movement of the hindlimbs; 21: normal movement; 0–7: little or no movement in the hindlimbs (early-recovery); 8–13: incoordination in walking (mid-recovery); 14–21: coordination between forelimbs and hindlimbs (recovered later)]. BBB scores were performed 12 h, 1 d, 3 d, 7 d, and 14 d after SCI surgery.

### Real-time quantitative PCR analysis

Total RNA was extracted from tissue samples and PC12 cells with the TRIzol (Vazyme), and its concentration was measured by NanoDrop ND-1000 system (Thermo Fisher Scientific). Denaturing agarose gel electrophoresis was performed to assess the RNA integrity. The OD260/OD280 values ranging from 1.8 to 2.1 indicated that the RNA met the qualifications for purity. We used 1 μg of total RNA for reverse transcription with a PrimeScript RT Kit (TaKaRa). Real-time quantitative PCR (RT-qPCR) reactions were conducted with the AceQ Universal SYBR qPCR Master Mix (Vazyme). The relative expression was calculated using the 2^−ΔΔCt^ method with β-actin as the internal reference. The primers were used in this study as followed: β-actin primers: forward 5′-AGGCCAACCGTGAAAAGATG-3′, reverse 5′-ATGCCAGTGGTACGACCAGA-3′; Clec5a primers: forward 5′-TTCCCCATCCACCTACCTTC-3′, reverse 5′-GAGGAGGTTGTGAAGCCGAG-3′; Trem1 primers: forward 5′-AACCCGATCCCTACCCAAGT-3′, reverse 5′-GATGAGGAGCCCACAGACCA-3′; Nlrc4 primers: forward 5′-CACGGTGTGAGCAGTGATGG-3′, reverse 5′-CGCTGCGTCTGGTAAGAACTC-3′.

### Western blotting

Extraction of total protein was carried out with the RIPA lysis buffer (Thermo) containing protease inhibitors, and the concentration was assessed by the Pierce BCA Protein Assay Kit (Thermo). Thirty micrograms of proteins were electrophoresed in a 10% sodium dodecyl sulfate polyacrylamide gel and transferred onto nitrocellulose membrane. The membranes were incubated at 4°C for 12 h with the antibody against CLEC5A, TREM1, NLRC4, Caspase-1, IL-1β, GSDMD, ASC, IL-18, and GAPDH after blocking in 5% blocking buffer. The membranes were incubated for 2 h with the secondary antibody at room temperature. Proteins were visualized using enhanced chemiluminescence substrate (Thermo), and the blots were analyzed by ImageJ software (National Institutes of Health).

### Hematoxylin–eosin staining

Spinal cord samples were fixed in 4% paraformaldehyde for 24 h and then embedded in paraffin and sliced into 5-µm-thick sections. Slices were dewaxed, ethanol rehydrated, and then stained with hematoxylin–eosin (HE) to understand general morphological changes under an inverted microscope (IXplore Pro, OLYMPUS).

### Cell culture and treatment

PC12 cells (American Type Culture Collection) were cultured in Roswell Park Memorial Institute-1640 (RPMI-1640) medium (HyClone) containing 10% fetal bovine serum (Thermo) and 1% penicillin-streptomycin mixture (Solarbio) in humidified 5% CO_2_ at 37°C. For lidocaine treatment, different concentrations of lidocaine (0.1, 0.5, and 1 mM; Solarbio) were administered to PC12 cells.

### Immunofluorescence

The sections (30 µm) were washed with 0.5% Triton X-100 for 10 min, and blocked with 5% bovine serum albumin (Sigma) and 0.5% Triton X-100 (Sigma) for 1 h. The following primary antibodies were used: NeuN, caspase-1, IL-18, CLEC5A, and TREM1. Sections were then washed, followed by incubation with appropriate secondary antibodies conjugated to Alexa Fluor (Thermo). The sections were mounted and coverslipped in Vectashield mounting medium with DAPI (Vector Laboratories). The sections were observed with a fluorescence microscope (OLYMPUS) and quantitative analyzed by Fiji software (National Institutes of Health). The colocalization of CLEC5A and TREM1 was analyzed by Image-Pro Plus (Media Cybernetics).

### Tunel staining

Tunel costaining with NeuN was performed after SCI. After permeabilization with 0.1% PBS-Triton X-100 for 15 min, the sections were blocked in 10% bovine serum albumin for 1 h and then incubated with a primary antibody against NeuN (1:100) at 4°C overnight. After incubation with 0.1% goat anti-mouse IgG H&L in Tunel staining liquid for 1 h at room temperature, the images were observed by a microscope (OLYMPUS).

### Detection of lactate dehydrogenase release

Lactate dehydrogenase (LDH) levels were measured in the cell supernatant and serum of rats using an LDH assay kit (Beyotime) based on the manufacturer's instructions. Absorbance was detected at 490 nm using a microplate reader (Thermo).

### Enzyme-linked immunosorbent assay

The IL-1β (Beyotime) and IL-18 (Beyotime) levels in cell supernatant were measured using IL-1β and IL-18 Enzyme-linked immunosorbent assay (ELISA) kits according to the instructions. The absorbance at 450 nm was measured with a microplate reader (Thermo).

### 3-(4,5-Dimethylthiazol-2-yl)-2,5-diphenyltetrazolium bromide (MTT) assay

The viability of PC12 cells (1 × 10^4^cells/well) treated with different concentrations of lidocaine (0.1, 0.5, 1, 5, and 10 mM) was detected by the MTT solution (Aladdin). The MTT solution at a final concentration of 1 mg/ml was incubated with PC12 cells for 4 h at 37°C. Formazan crystal was dissolved with 150 μl/well of DMSO. Measurement of the absorbance at 490 nm was done with a microplate reader (Thermo).

### Cell pyroptosis detection

The Hoechst 33342/propidium iodide (PI) double fluorescence staining kit (CA1120, Solarbio) was used for pyroptosis detection. PC12 cells were seeded into 96-well plates (1 × 10^5^ cells/well) and treated with lidocaine 1 (nM) for 24 h. Approximately 1 × 10^4^ cells were trypsinized and resuspended in 1× binding buffer, followed by staining with Hoechst 33342 solution (10 μl) for 10 min under dark conditions. The results were observed using a fluorescence microscope (OLYMPUS) after staining with 5 μl PI in the dark for 15 min.

### Co-immunoprecipitation

The lysates of PC12 cells obtained by lysis with precooled RIPA lysis solution were added with anti-CLEC5A, anti-TREM1, anti-NLRC4, or IgG antibodies (1:100, Abcam), and the antigen–antibody mixtures were slowly shaken at 4°C overnight on a shaker. Then, protein A/G agarose beads (100 μl) were added to capture the antigen–antibody complexes. After washing with precooled RIPA buffer, the complexes were suspended with 2× loading buffer and then boiled for 5 min. The obtained supernatant was subjected to Western blotting.

### Statistical analysis

Data were statistically analyzed using GraphPad 8.0 software and summarized as mean ± standard deviation. Comparisons between two independent groups were performed using a two-tailed unpaired *t* test. Analysis of variance (ANOVA) with Tukey's multiple-comparisons test was used to analyze differences among three or more groups. Statistical results are considered significant if *p *< 0.05.

## Results

### CLEC5A, TREM1, and NLRC4 were highly expressed in lidocaine-induced SCI rat models, and their knockdown alleviated lidocaine-induced SCI

**Figure 1.**
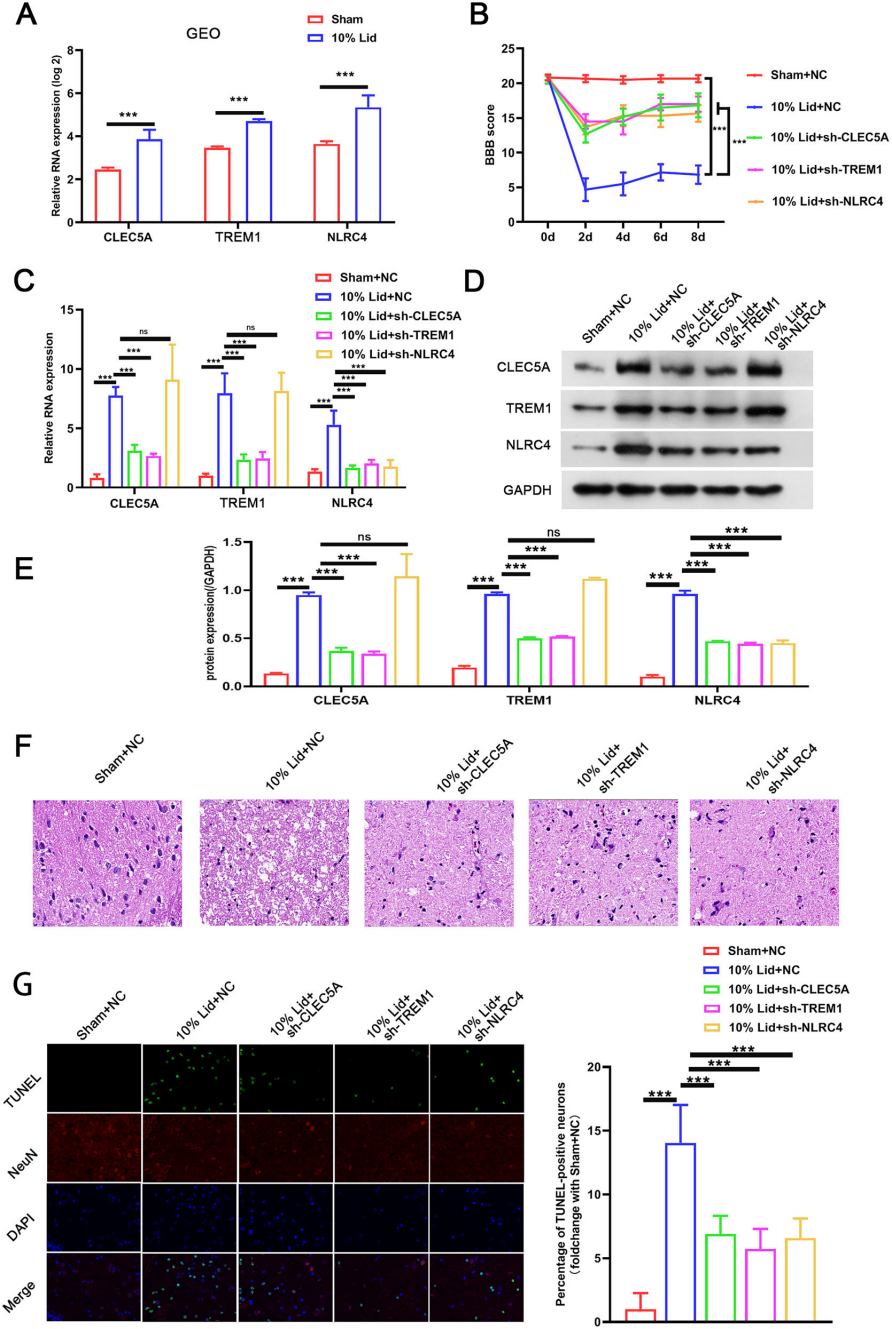
CLEC5A, TREM1, or NLRC4 knockdown mitigated lidocaine-induced SCI. ***A***, Data on the expression patterns of CLEC5A, TREM1, and NLRC4 in the lidocaine-induced SCI came from GSE136833. Comparisons between two independent groups were performed using a two-tailed unpaired *t* test. ***B***, Analysis of rat behavioral score was performed using the BBB scale. ***C–E***, Detection of CLEC5A, TREM1, or NLRC4 mRNA and protein levels in spinal cord samples was carried out by RT-qPCR and Western blotting, respectively. ***F***, Evaluation of pathological injury status of rat spinal cord tissues was performed using HE staining. ***G***, Observation of the apoptosis of neurons in spinal cord samples by costaining with tunel and NeuN. Data are mean ± standard deviation (SD; *n* = 6). ***p < 0.001. NS, not significant. Analysis of variance (ANOVA) with Tukey's multiple-comparisons test was used to analyze differences among three or more groups.

Higher levels of CLEC5A, TREM1, and NLRC4 were found in the lidocaine-induced SCI in the GSE136833 database (Fig. 1*A*). Here, elucidation of the roles of CLEC5A, TREM1, and NLRC4 in SCI was performed by intrathecal injection of lentivirus-mediated shCLEC5A, shTREM1, or shNLRC4 into SCI rat models. Evaluation results of the BBB scale showed that lidocaine damaged the activity of rats and reduced BBB scores compared with the sham group, indicating the successful construction of SCI induced by lidocaine in rats. Compared with the lidocaine group rats, the BBB scores of the lidocaine + shCLEC5A/shTREM1/shNLRC4 group were significantly elevated, indicating that knockdown of CLEC5A, TREM1, or NLRC4 could ease SCI in rats (Fig. 1*B*). Next, the observation of the changes in the levels of CLEC5A, TREM1, and NLRC4 in spinal cord samples showed that CLEC5A, TREM1, and NLRC4 had higher mRNA and protein levels in the lidocaine group compared with the sham group, while the mRNA and protein levels of CLEC5A, TREM1, and NLRC4 were downregulated in the lidocaine + shCLEC5A/shTREM1/shNLRC4 group compared with the lidocaine group (Fig. 1*C–E*). The pathological changes in the spinal cord tissue of the lidocaine group rats mainly showed interstitial edema, local vacuoles, and neuronal degeneration and consolidation, while these pathological changes were significantly alleviated in the lidocaine + shCLEC5A/shTREM1/shNLRC4 group (Fig. 1*F*). Furthermore, the lidocaine group had a large distribution of tunel/NeuN-positive cells in the spinal cord samples, while the lidocaine + shCLEC5A/shTREM1/shNLRC4 group had a smaller number of tunel/NeuN-positive cells compared with the lidocaine group, indicating that knockdown of CLEC5A, TREM1, or NLRC4 weakened neuronal apoptosis in SCI models, which was confirmed by immunofluorescence (Fig. 1*G*). All results manifested that CLEC5A, TREM1, or NLRC4 silencing could alleviate lidocaine-induced SCI in rat models.

### Knockdown of CLEC5A, TREM1, or NLRC4 lightened neuronal pyroptosis in lidocaine-induced SCI rats

**Figure 2.**
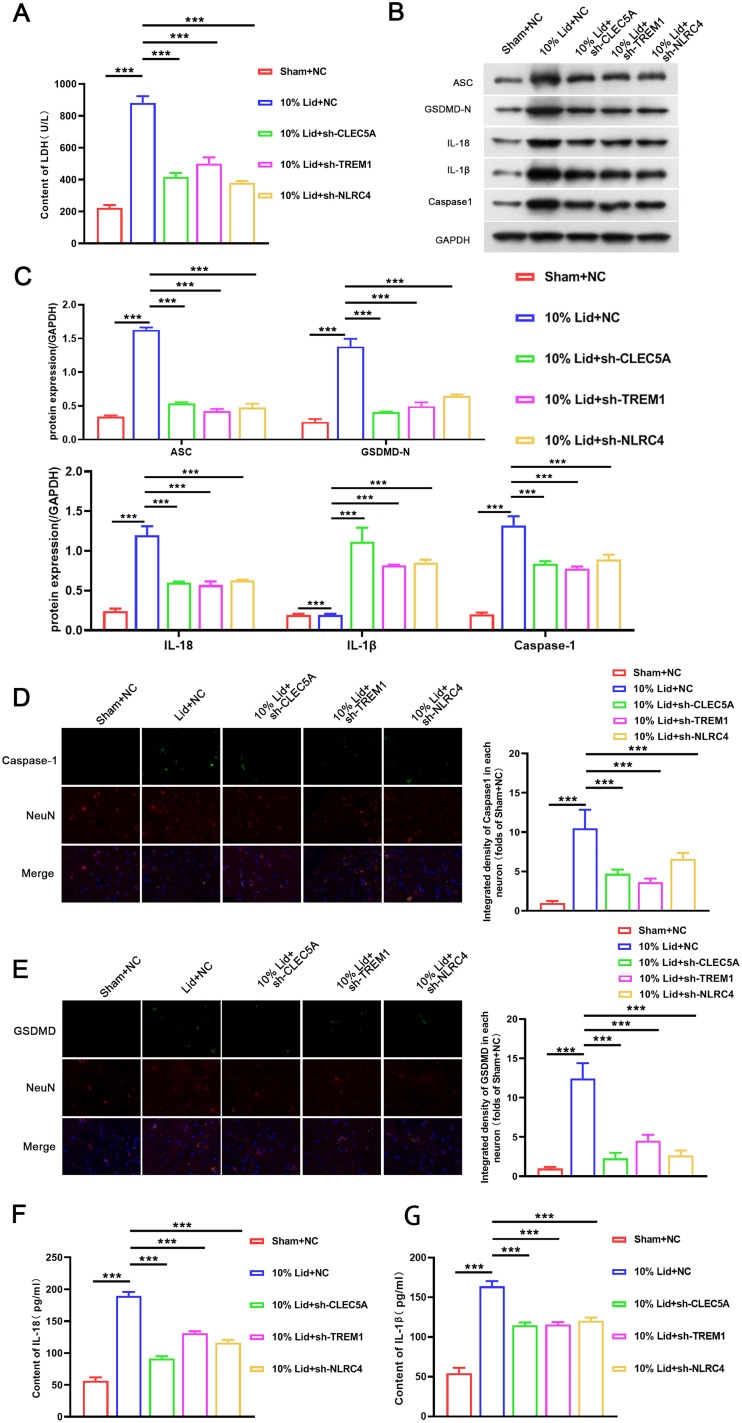
Inhibition of CLEC5A, TREM1, or NLRC4 undercut neuronal pyroptosis in lidocaine-induced SCI rats. ***A***, The LDH levels in the serum of rats from the sham + NC, lidocaine + NC, lidocaine + shCLEC5A, lidocaine + shTREM1, and lidocaine + shNLRC4 groups were analyzed using a LDH kit. ***B***, ***C***, Protein levels of ASC, GSDMD-N, IL-18, caspase-1, and IL-1β in rat spinal cord samples from different groups was evaluated by Western blotting. ***D***, ***E***, Immunofluorescence staining for NeuN/caspase-1 and NeuN/IL-18 colocalization was conducted to analyze the pyroptosis of neurons in rat spinal cord samples from different groups. ***F***, ***G***, ELISA was utilized for analysis of the serum concentrations of IL-18 and IL-1β in rats from different groups. Data are mean ± SD (*n* = 6). ***p < 0.001. ANOVA with Tukey's multiple-comparisons test was used to analyze differences among three or more groups.

Based on the crucial role of pyroptosis in the process of SCI, we analyzed the effects of CLEC5A, TREM1, or NLRC4 on neuronal pyroptosis in rat SCI models. We observed that lidocaine resulted in a significant increase in LDH content compared with the sham group, but inhibition of CLEC5A, TREM1, or NLRC4 impaired the increased LDH content induced by lidocaine (Fig. 2*A*). Moreover, higher protein levels of ASC, GSDMD-N, IL-18, caspase-1, and IL-1β were detected in lidocaine-induced SCI rat-derived spinal cord samples compared with the sham group rat-derived spinal cord samples. And the increased protein levels of ASC, GSDMD-N, IL-18, caspase-1, and IL-1β in lidocaine-induced rat spinal cord samples were weakened after CLEC5A, TREM1, or NLRC4 silencing (Fig. 2*B*,*C*). Furthermore, immunofluorescence staining for NeuN/caspase-1 and NeuN/GSDMD-N colocalization in spinal cord samples showed the cellular localization of neurons with pyroptosis after lidocaine treatment. However, silencing of CLEC5A, TREM1, or NLRC4 lightened the pyroptosis of neurons in lidocaine-mediated rat spinal cord samples (Fig. 2*D*,*E*). The serum concentrations of IL-18 and IL-1β were higher in SCI rats than in sham rats. However, the elevated serum concentrations of IL-18 and IL-1β were attenuated in SCI rats after silencing of CLEC5A, TREM1, or NLRC4 (Fig. 2*F*,*G*). Together, CLEC5A, TREM1, or NLRC4 downregulation lightened neuronal pyroptosis in lidocaine-induced SCI rats.

### Knockdown of CLEC5A, TREM1, or NLRC4 alleviated lidocaine-induced PC12 cell pyroptosis

**Figure 3.**
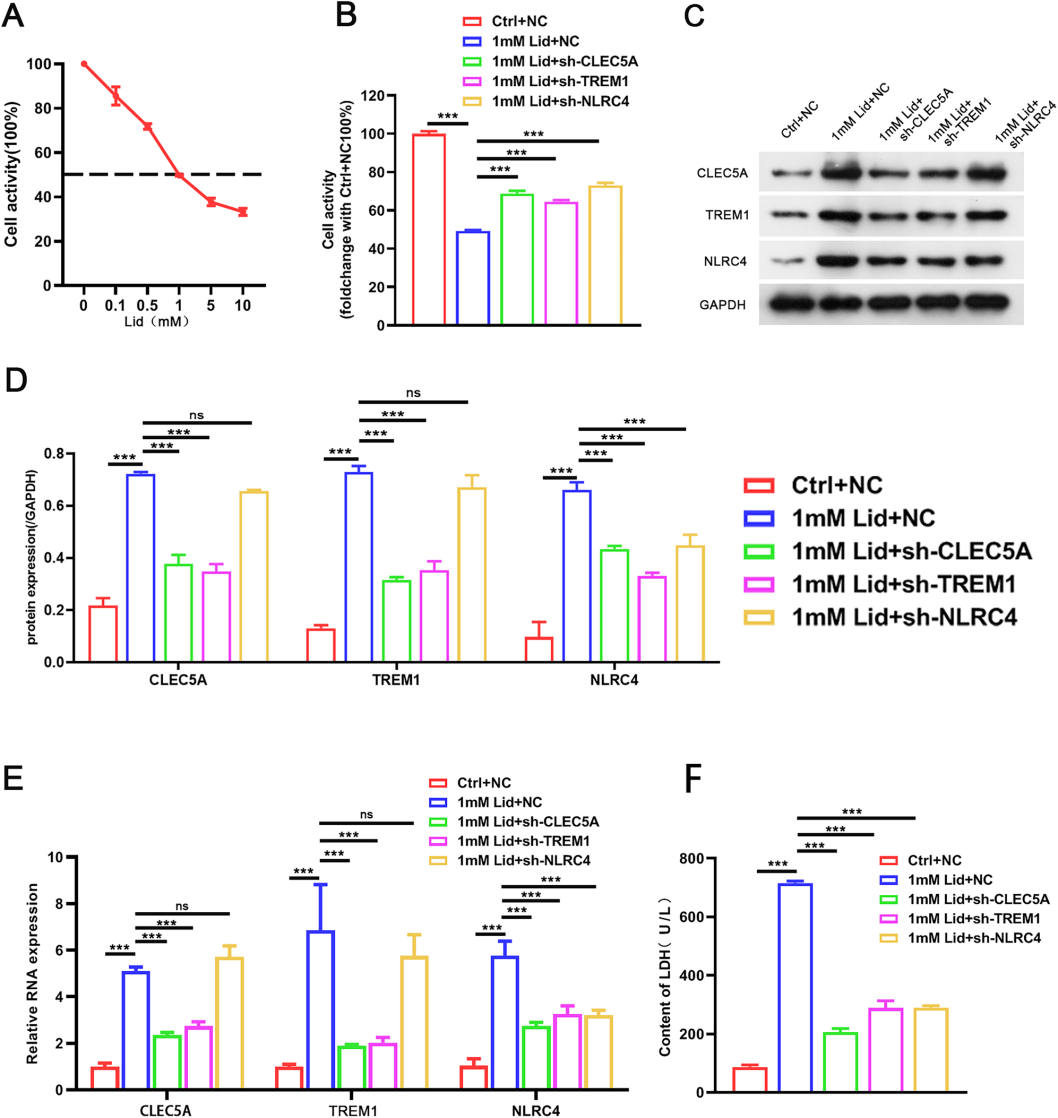
Downregulation of CLEC5A, TREM1, or NLRC4 elevated PC12 cell viability under lidocaine treatment. ***A***, The viability of PC12 cells stimulated with different concentrations of lidocaine (0, 0.1, 0.5, 1, 5, and 10 nM) was determined by MTT assays. ***B***, The viability of the shCLEC5A-, shTREM1-, or shNLRC4-infected PC12 cells treated with lidocaine. ***C–E***, Relative mRNA and protein levels of CLEC5A, TREM1, or NLRC4 in the shCLEC5A-, shTREM1-, or shNLRC4-infected PC12 cells treated with lidocaine. ***F***, Assessment of cell damage in different groups was made by detection of LDH release. Data are mean ± SD (*n* = 3). ***p < 0.001. NS, not significant. ANOVA with Tukey's multiple-comparisons test was used to analyze differences among three or more groups.

**Figure 4.**
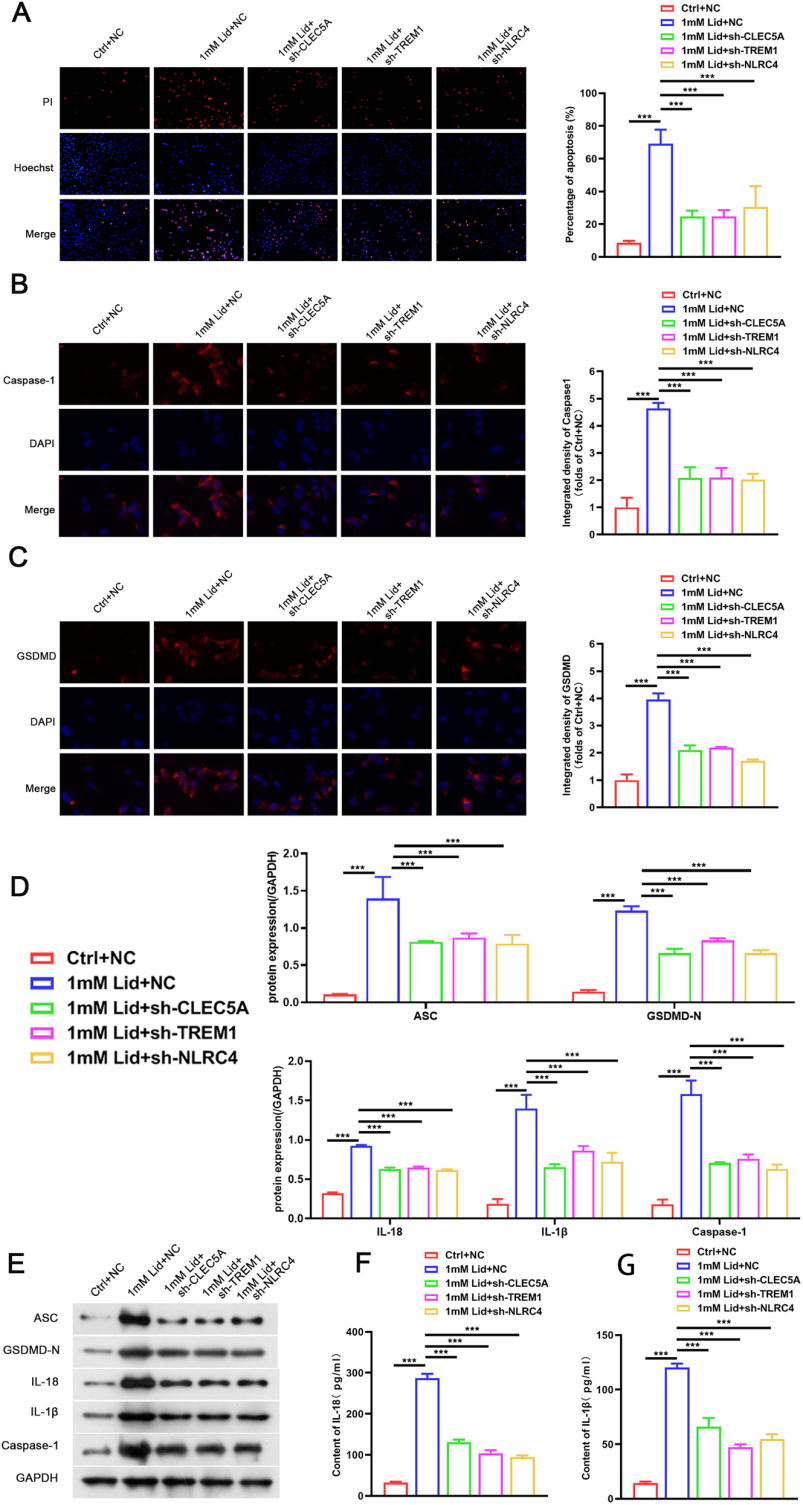
Silencing of CLEC5A, TREM1, or NLRC4 lessened PC12 cell pyroptosis under lidocaine treatment. ***A–C***, The number of PI, caspase-1, and GSDMD-N-positive cells in the shCLEC5A-, shTREM1-, or shNLRC4-infected PC12 cells treated with lidocaine was analyzed by immunofluorescence. ***D***, ***E***, Protein levels of ASC, GSDMD-N, IL-18, caspase-1, and IL-1β in PC12 cells of different groups. ***F***, ***G***, The release of IL-18 and IL-1β from PC12 cells into the supernatant was evaluated by ELISA. Data are mean ± SD (*n* = 3). ***p < 0.001. ANOVA with Tukey's multiple-comparisons test was used to analyze differences among three or more groups.

Subsequently, we further elucidated the effect of CLEC5A, TREM1, or NLRC4 on neuronal pyroptosis in vitro by constructing lidocaine-induced PC12 cells. Lidocaine had inhibitory effects on PC12 cells in a concentration-dependent manner, and its IC50 was used for subsequent analysis (Fig. 3*A*). We also observed that the inhibitory effect of lidocaine on PC12 cell viability was partly reversed after CLEC5A, TREM1, or NLRC4 silencing (Fig. 3*B*). Furthermore, the mRNA and protein levels of CLEC5A, TREM1, or NLRC4 were overexpressed in PC12 cells treated with lidocaine, but the elevated CLEC5A, TREM1, or NLRC4 levels mediated by lidocaine were weakened after CLEC5A, TREM1, or NLRC4 downregulation (Fig. 3*C–E*). Evaluation of LDH release and PI staining was made to analyze cell death. The results showed that lidocaine accelerated LDH release in the supernatant and increased the number of PI-positive PC12 cells, but these changes were partly overturned following CLEC5A, TREM1, or NLRC4 knockdown (Figs. 3*F*, 4*A*). In addition, lidocaine led to the elevation in the number of caspase-1 and GSDMD-N-positive cells, but these elevated caspase-1 and GSDMD-N-positive cells were weakened by CLEC5A, TREM1, or NLRC4 inhibition (Fig. 4*B*,*C*). As shown in Figure 4*D*,*E*, treatment with lidocaine markedly elevated ASC, GSDMD-N, IL-18, caspase-1, and IL-1β protein levels in PC12 cells, while these elevated protein levels were impaired by knockdown of CLEC5A, TREM1, or NLRC4. Similarly, the promoting effect of lidocaine on the release of IL-18 and IL-1β from PC12 cells into the supernatant was lowered by downregulating CLEC5A, TREM1, or NLRC4 (Fig. 4*F*,*G*). All in all, downregulation of CLEC5A, TREM1, or NLRC4 could reduce lidocaine-induced PC12 pyroptosis.

### CLEC5A interacted with TREM1 to mediate NLRC4 expression

**Figure 5.**
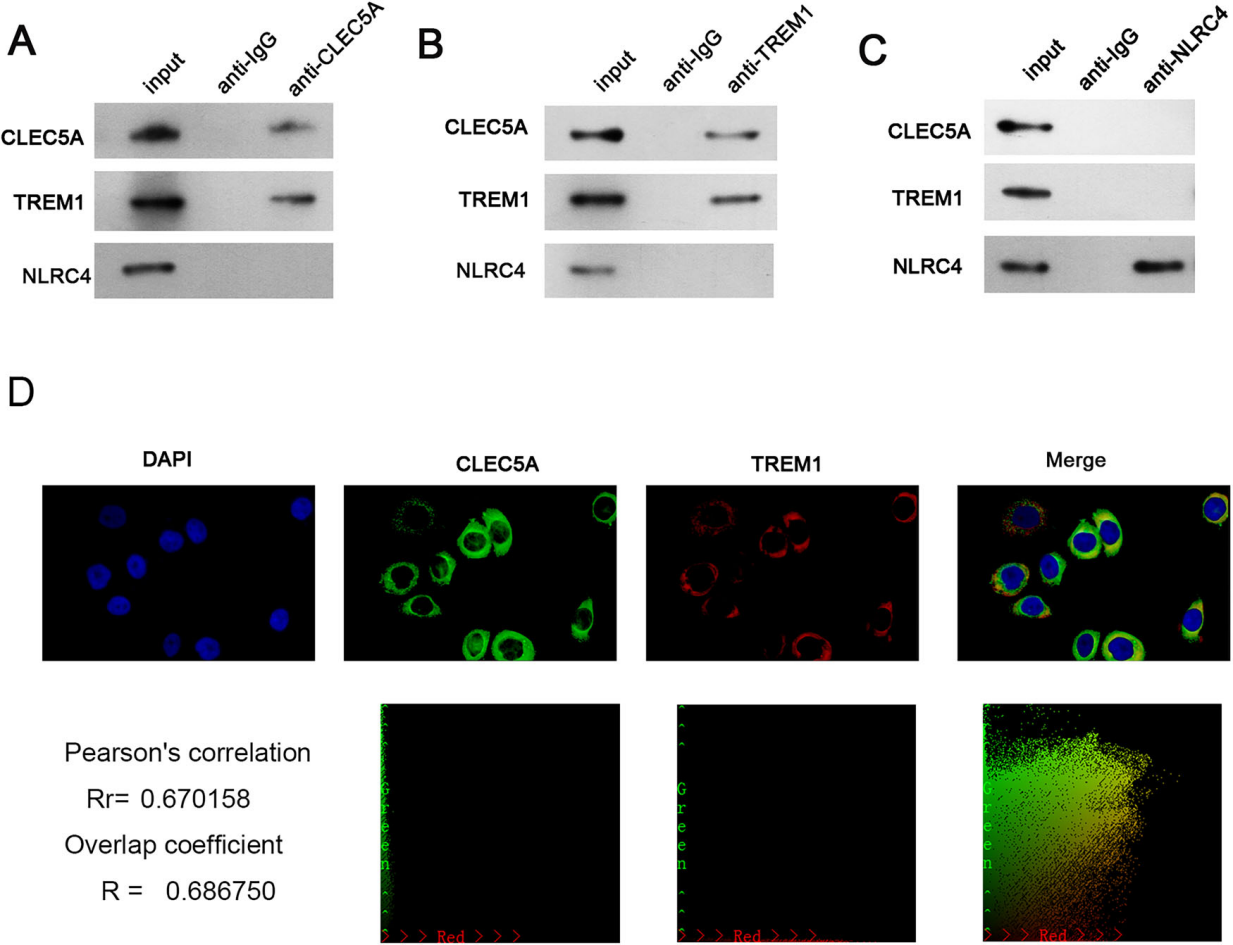
CLEC5A interacted with TREM1 in PC12 cells. ***A–C***, Co-IP assay using antibody against CLEC5A, TREM1, or NLRC4 as bait protein demonstrated the interaction between among CLEC5A, TREM1, and NLRC4. ***D***, Sublocalizations of CLEC5A and TREM1 were analyzed using immunofluorescence. Data are mean ± SD (*n* = 3).

It has been confirmed that both CLEC5A and TREM1 can regulate NLRP3 (Liang et al., 2020; Liu et al., 2021). NLRC4 and NLRP3 are both members of the NLRs family (H. Chen et al., 2020), and we speculated that TREM1 and CLEC5A may be able to regulate the expression of NLRC4. Further documentation of the interaction between TREM1 and CLEC5A was obtained with co-immunoprecipitation (Co-IP). Endogenous TREM1 was immunoprecipitated from lidocaine-treated PC12 cell lysates by anti-CLEC5A antibody, but not NLRC4 (Fig. 5*A*). The interaction was further confirmed by immunoprecipitation of CLEC5A with anti-TREM1 antibody (Fig. 5*B*). Neither endogenous TREM1 nor CLEC5A could be immunoprecipitated from lidocaine-treated PC12 cell lysate by anti-NLRC4 antibody (Fig. 5*C*). We further confirmed the interaction between TREM1 and CLEC5A by immunofluorescence analysis. The results exhibited that CLEC5A predominantly colocalized with TREM1 in lidocaine-treated PC12 cells (Fig. 5*D*). Importantly, CLEC5A knockdown decreased TREM1 and NLRC4 mRNA and protein levels in SCI rat spinal cord samples and PC12 cells stimulated with lidocaine, and both TREM1 and CLEC5A knockdown decreased the mRNA and protein levels of NLRC4 in SCI rat spinal cord samples and PC12 cells stimulated with lidocaine (Figs. 1*D*, 3*C*,*D*). The above results exhibited that CLEC5A interacted with TREM1 to regulate NLRC4 expression.

### CLEC5A mediated NLRC4 expression by interacting with TREM1, promoting PC12 cell pyroptosis under lidocaine treatment

**Figure 6.**
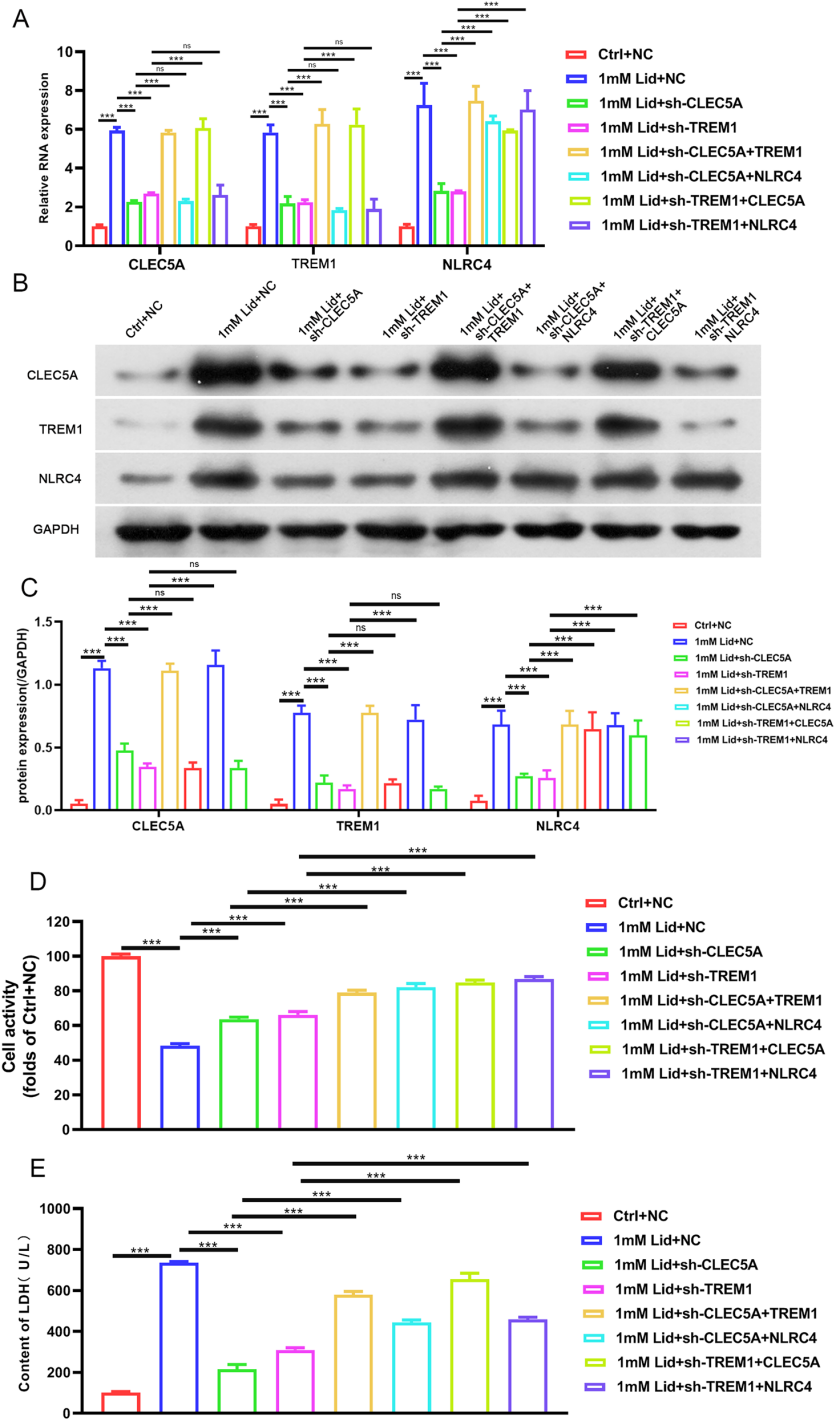
CLEC5A promoted lidocaine-induced PC12 cell viability by interacting with TREM1 and regulating NLRC4 expression. ***A–C***, Relative mRNA and protein levels of CLEC5A, TREM1, or NLRC4 in the shCLEC5A-, shTREM1-, or shNLRC4-infected PC12 cells treated with lidocaine and CLEC5A, TREM1, or NLRC4 plasmid. ***D***, The viability of PC12 cells was determined by MTT assays in different groups. ***E***, Cell damage in different groups was assessed by detection of LDH release. Data are mean ± SD (*n* = 3). ***p < 0.001*.* NS, not significant. ANOVA with Tukey's multiple-comparisons test was used to analyze differences among three or more groups.

We further verified whether CLEC5A is involved in lidocaine-mediated PC12 cell pyroptosis through its interaction with TREM1 to mediate the expression of NLRC4. The results exhibited that either CLEC5A or TREM1 knockdown weakened the promoting effect of lidocaine on CLEC5A, TREM1, and NLRC4 mRNA and protein levels. Introduction of TREM1 reversed CLEC5A knockdown-mediated effects on CLEC5A, TREM1, and NLRC4 mRNA and protein levels in lidocaine-stimulated PC12 cells. NLRC4 overexpression only attenuated the effect of CLEC5A knockdown on NLRC4 but had no effect on CLEC5A and TREM1. In addition, TREM1 knockdown-mediated effects on CLEC5A, TREM1, and NLRC4 levels were impaired by CLEC5A overexpression. Similarly, exogenous expression of NLRC4 only lessened the effect of TREM1 inhibition on NLRC4 but did not affect on CLEC5A and TREM1 (Fig. 6*A–C*). These results manifested that CLEC5A regulated NLRC4 expression through interacting with TREM1.

**Figure 7.**
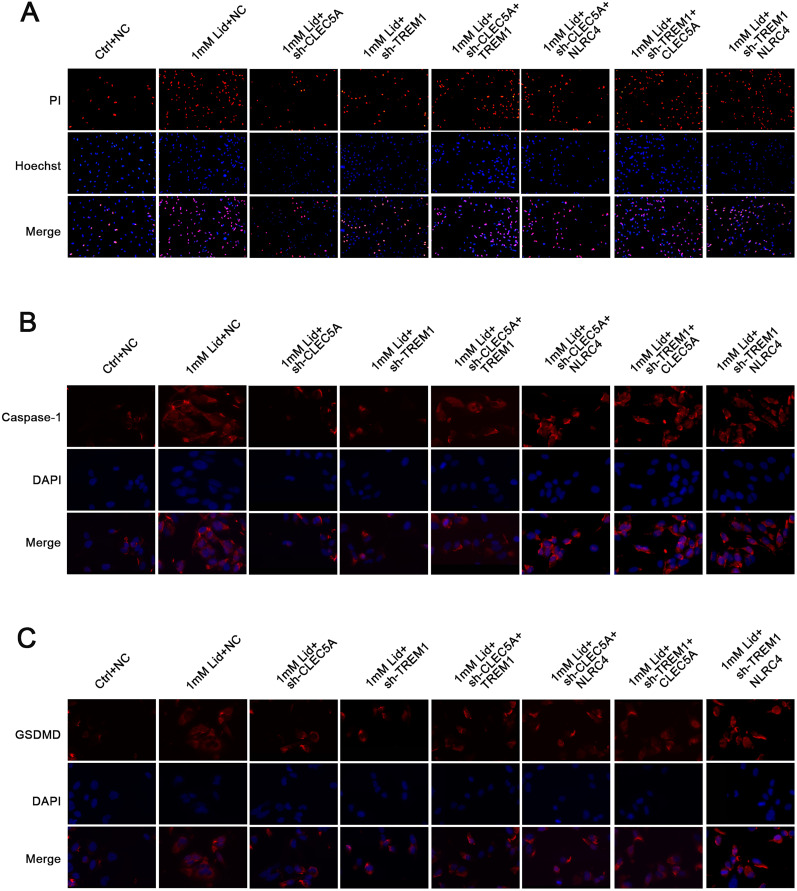
CLEC5A promoted PC12 cell pyroptosis by mediating NLRC4 expression by interacting with TREM1. ***A–C***, Immunofluorescence analysis of the number of PI, caspase-1, and GSDMD-N-positive cells in the shCLEC5A-, shTREM1-, or shNLRC4-infected PC12 cells treated with lidocaine and CLEC5A, TREM1, or NLRC4 plasmid. Data are mean ± SD (*n* = 3).

**Figure 8.**
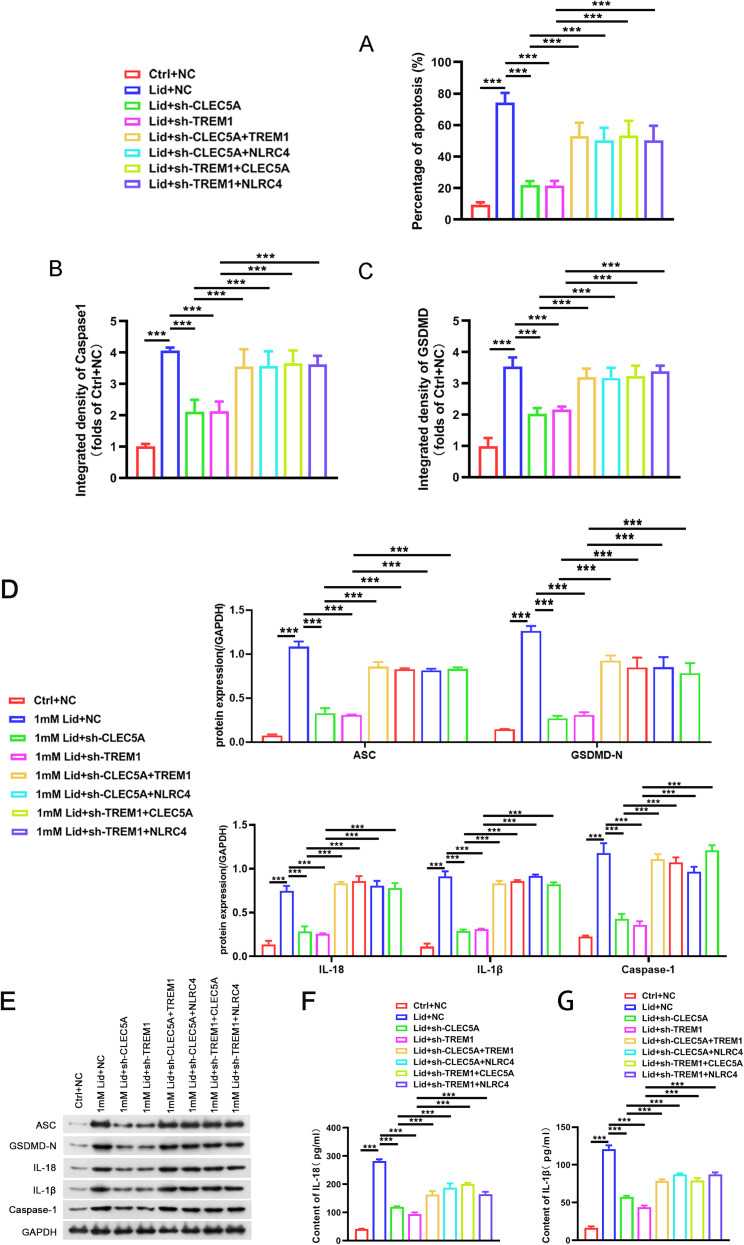
CLEC5A promoted PC12 cell pyroptosis by mediating NLRC4 expression by interacting with TREM1. The quantification of PI (***A***), caspase-1 (***B***), and GSDMD-N (***C***)-positive cells in the shCLEC5A-, shTREM1-, or shNLRC4-infected PC12 cells treated with lidocaine and CLEC5A, TREM1, or NLRC4 plasmid. ***D***, ***E***, Assessment of protein levels of ASC, GSDMD-N, IL-18, caspase-1, and IL-1β was done in the shCLEC5A-, shTREM1-, or shNLRC4-infected PC12 cells treated with lidocaine and CLEC5A, TREM1, or NLRC4 plasmid. ***F***, ***G***, ELISA determined the release of IL-18 and IL-1β from PC12 cells into the supernatant indifferent groups. Data are mean ± SD (*n* = 3). ***p < 0.001. ANOVA with Tukey's multiple-comparisons test was used to analyze differences among three or more groups.

We also observed that both TREM1 and NLRC4 overexpression reversed CLEC5A silencing-medicated effects on cell viability and death in lidocaine-stimulated PC12 cells. Similarly, TREM1 inhibition-medicated effects on cell viability and death could be partly overturned by overexpression of CLEC5A or TREM1 (Figs. 6*D*,*E*, 7*A*). Also, upregulation of TREM1 or NLRC4 reversed the effect of CLEC5A inhibition on caspase-1 and GSDMD-N-positive cells, ASC, GSDMD-N, IL-18, caspase-1, and IL-1β protein levels and the release of IL-18 and IL-1β (Figs. 7*B*,*C*, 8*A–G*). As expected, CLEC5A or NLRC4 upregulation overthrew the effect of TREM1 silencing on caspase-1 and IL-18-positive cells, ASC, GSDMD-N, IL-18, caspase-1, and IL-1β protein levels and the release of IL-18 and IL-1β (Figs. 7*B*,*C*, 8*A–F*). Altogether, CLEC5A interacted with TREM1 to regulate NLRC4 expression, thus promoting lidocaine-induced PC12 cell pyroptosis.

## Discussion

Pyroptosis, as an important innate immune mechanism in vertebrates, is another important way of programmed cell death besides apoptosis (Bertheloot et al., 2021). Activation of inflammasomes plays an important role in the process of cell pyroptosis. Numerous studies have shown that pyroptosis-related molecules are upregulated in SCI animal models, and inhibiting pyroptosis to alleviate neuroinflammation will be a new approach to treating SCI (Liu et al., 2021; C. Wu et al., 2021). CLEC5A, also known as MDL-l, is a recognized receptor for certain pathogenic microorganisms (Sung et al., 2022). An increasing amount of evidence suggests that CLEC5A has critical functions in various diseases, such as gastric cancer and chronic obstructive pulmonary disease (Q. Wang et al., 2020; Tosiek et al., 2022). Previous studies have confirmed a close relationship between CLEC5A and inflammatory response (Fu et al., 2021; Machado et al., 2023). In addition, CLEC5A has been uncovered to exert an important role in NLRP3 inflammasome activation and pyroptosis in myocardial infarction-induced cardiac dysfunction (X. Wang et al., 2021) and human macrophages (M. F. Wu et al., 2013). However, it is unclear whether CLEC5A can mediate the pyroptosis of neurons to participate in SCI.

The destruction of cell membranes by Gasdermin protein is the key to pyroptosis (Mulla et al., 2023). At present, the Gasdermin family proteins found to be involved in pyroptosis mainly include Gasdermin (GSDM) A, GSDMB, GSDMC, GSDMD, and GSDME (H. Wang et al., 2023). Among them, GSDMD expression is most common in SCI (S. Xu et al., 2021; D. Zhang et al., 2023). Under normal conditions, the GSDMD protein is in an inactive state due to the presence of its N-terminal and C-terminal domains (Kong and Zhang, 2023). However, GSDMD can be activated through hydrolysis and cleavage, resulting in loss of cell membrane integrity, which can cause increased intracellular osmotic pressure, resulting in cell swelling, lysis, and death (Kamajaya and Boucher, 2022). The signaling molecules that cause GSDMD activation are mainly inflammatory caspases. Among them, caspase-1 is involved in the formation of inflammasome complexes and mediates pyroptosis (Xiaodong and Xuejun, 2023). Here, we found that CLEC5A was highly expressed in lidocaine-induced SCI models through biological information analysis (GSE136833 database). Further experiments demonstrated the upregulation of CLEC5A in lidocaine-induced SCI rat models, and CLEC5A knockdown alleviated lidocaine-induced SCI, indicating that CLEC5A was associated with SCI. In addition, CLEC5A inhibition weakened the colocalization of NeuN/caspase-1 and NeuN/IL-18 as well as decreased protein levels of ASC, GSDMD-N, IL-18, caspase-1, and IL-1β in lidocaine-induced rat spinal cord samples, suggesting that CLEC5A knockdown decreased the pyroptosis of neurons in lidocaine-induced rat SCI models. What's more, silencing of CLEC5A lessened lidocaine-induced PC12 cell pyroptosis. All data taken together indicated that CLEC5A could exacerbate SCI by promoting neuronal pyroptosis.

TREM1 is a surface-activated receptor expressed on neutrophils, monocyte, and macrophages (C. Zhang et al., 2022). According to reports, TREM1 may serve as a prognostic factor for cancer prevention (Pu et al., 2023). TREM1 could aggravate neuro-inflammatory injury (P. Xu et al., 2021) and septic cardiomyopathy (Yang et al., 2023) via activation of NLRP3 inflammasome-mediated pyroptosis. TREM1 also impaired cerebral ischemia-induced neuronal injury by inhibiting pyroptosis by targeting LP17 (Liang et al., 2020). In addition, TREM1 is a key target for neuronal apoptosis in the spinal cord, and downregulation of TREM1 might have a positive effect on the treatment of SCI-RI (W. Shi et al., 2021). Moreover, inhibition of TREM1 significantly improved the results of SCI by reducing inflammation and oxidative stress (Li et al., 2019). It was reported that TREM1 was a differently expressed gene in lidocaine-induced SCI models (GSE136833 database). Our data also showed the overexpression of TREM1 in lidocaine-induced SCI models. Moreover, TREM1 silencing decreased neuronal pyroptosis in lidocaine-induced SCI rat models and lidocaine-induced PC12 cells. Notably, the interaction between TREM1 and CLEC5A was verified in lidocaine-induced PC12 cells, and CLEC5A mediated lidocaine-induced PC12 cell pyroptosis by interacting with TREM1.

Previous studies have confirmed that both C LEC5A and TREM1 can regulate NLRP3 (L. Wang et al., 2021; X. Wang et al., 2021). NLRC4 and NLRP3 are both members of the NLRs family (H. Chen et al., 2020). NLRC4 inflammasome is an important receptor involved in immune response, which can cause inflammatory reaction (Egan et al., 2023). Multiple studies have shown that NLRC4 inflammasome plays an important role in various inflammatory-related diseases, so inhibiting the activation of NLRC4 inflammasome may be a potential target for treating inflammatory-related diseases (Davari et al., 2023; Deng et al., 2023; Gao et al., 2023). SMS1 triggered nonalcoholic steatohepatitis by regulating NLRC4-dependent hepatocyte pyroptosis (Koh et al., 2021). NLRC4 and inflammatory cytokines associated with pyroptosis taken part in diabetic cerebral ischemia-reperfusion injury (L. Q. Wang et al., 2021). Furthermore, cerebral hemorrhage-mediated damage had been discovered to be related to NLRC4-dependent neuronal pyroptosis (Jin et al., 2022). Our research verified that NLRC4 was a downstream gene of CLEC5A and TREM1 and CLEC5A could regulate NLRC4 expression by interacting with TREM1. In addition, CLEC5A interacted with TREM1 to regulate NLRC4 expression, thus promoting lidocaine-induced neuronal pyroptosis in PC12 cells.

### Conclusion

Taken together, our study revealed a new mechanism of pyroptosis related to neuronal death after SCI. Attentively, CLEC5A interacted with TREM1 to elevate NLRC4 expression, thus promoting neuronal pyroptosis in SCI rat models. Our research provides new insights into the role of neuronal pyroptosis in SCI.

## Data Availability

The original contributions presented in the study are included in the article; further inquiries can be directed to the corresponding author.

## Synthesis

Reviewing Editor: Jennifer Dulin, Texas A&M University Decisions are customarily a result of the Reviewing Editor and the peer reviewers coming together and discussing their recommendations until a consensus is reached. When revisions are invited, a fact-based synthesis statement explaining their decision and outlining what is needed to prepare a revision will be listed below. The following reviewer(s) agreed to reveal their identity: Kate O'Neill.

The reviewer and editor found the study to be of high quality and potentially suitable for publication in eNeuro, provided the following revisions are made:

In this manuscript, the authors investigate the mechanisms behind neuronal pyroptosis using an in vivo model of lidocaine-induced spinal cord injury and the PC12 cell line. The authors characterize the effects of CLEC5A, TREM1, and NLRC4 expression on spinal cord injury outcomes and also probe their interactions. Importantly, they find an interaction between CLEC5A and TREM1 that promotes neuronal pyroptosis through regulation of NLRC4 expression.

Overall, this is a nice study that, in my opinion, requires relatively few changes prior to publication.

Major concerns:

-- The Western blotting results are not quantified. Only representative images/blots are displayed. Please quantify the blots.

-- For the in vivo work, the authors mention that N=6 for each condition. What about for the in vitro work involving PC-12 cells? Please provide detailed information about experimental replicates for the PC-12 data in Figs. 3-7.

-- No analysis is provided for Fig. 5D. Please perform some type of co-localization analysis for the representative images shown in Fig. 5D.

Minor concners:

-- Fig. 2 is labeled incorrectly according to the figure legend. The Western blotting results are part B, not part D. A description of part F is missing in the legend.

-- Fig. 3 is also labeled incorrectly according to the figure legend. In the figure, part B quantifies the MTT assay, part C is the representative Western blot, and D quantifies mRNA expression.

-- Replot data to include raw datapoints. If in some figures the datapoints are too small to be legible (I expect this might happen in Fig. 7, for example), please include the raw data as supplementary figures.

-- Provide Ns for all figure parts in legends.

-- The statistical analysis appears correct and is mentioned in the methods. However, I request that the authors add the details regarding which tests were used to the figure legends.

-- In some places in the manuscript, the authors write "western blotting". The "W" should be capitalized to be "Western blotting".

## Author Response

We would like to thank you and the reviewers for their instructive comments and contributions to our manuscript (NO. eN-NWR-0111-24R1). We have revised the manuscript according to the reviewers' suggestions. The changes to the revised manuscript are indicated using the "Track Changes" function in Word, as requested. We have also responded to the reviewers' comments as detailed below in red.

- References must be in alphabetical order Response: The references have been arranged in alphabetical order
